# 4139 Risk Aversion in Lung Transplantation: Organ Procurement Organizations Differ in Willingness to Pursue Non-ideal Donor Organs

**DOI:** 10.1017/cts.2020.425

**Published:** 2020-07-29

**Authors:** Samantha Ellen Halpern, Alec McConnell, Sarah Peskoe, Vignesh Raman, Oliver K Jawitz, Ashley Y Choi, John C Haney, Jacob A Klapper, Matthew G Hartwig

**Affiliations:** 1 Duke University

## Abstract

OBJECTIVES/GOALS: Lung transplant (LTx) candidates benefit from use of non-ideal donor organs. Each organ procurement organization (OPO) defines “acceptable” donor organs introducing unmeasured variation in donor pursuit. We characterized non-ideal donor pursuit among OPOs to identify drivers of risk aversion in LTx. METHODS/STUDY POPULATION: We queried the UNOS registry for adult donors who donated ≥1 organ for transplantation from 12/2007-12/2018. Non-ideal donors were those with any of age>50, smoking history ≥20 pack-years, PaO_2_/FiO_2_ (P/F) ratio<350, donation after cardiac death (DCD) status, or CDC increased risk (IRD) status. Non-ideal donor pursuit rate was defined as the proportion of non-ideal donors at each OPO from whom consent for lung donation was requested with lower numbers indicating increased risk aversion. We estimated the correlation between non-ideal and overall donor pursuit using a Spearman correlation coefficient. Adjusted non-ideal donor pursuit rates were estimated using multivariable logistic regression. RESULTS/ANTICIPATED RESULTS: Overall, 18,333 deceased donors were included and classified as ideal or non-ideal. Among 58 OPOs, rates of non-ideal donor pursuit ranged from 0.24-1.00 **Figure**). Of 5 non-ideal characteristics, DCD and IRD status were associated with the most and least risk aversion, respectively. Non-ideal donor pursuit was strongly correlated with overall donor pursuit (r = 0.99). On adjusted analysis, older age (OR 0.15, 95% CI 0.13-0.16), smoking history (OR 0.38, 95% CI 0.34-0.44), low P/F ratio (OR 0.12, 95% CI 0.11-0.14), and DCD status (OR 0.04, 95% CI 0.03-0.04) were all independently associated with significant risk aversion, corresponding to decreased rates of donor pursuit. DISCUSSION/SIGNIFICANCE OF IMPACT: OPOs differ in their levels of risk aversion in LTx and risk aversion is not uniform across selected categories of non-ideal lung donor. Consideration of new OPO performance metrics that encourage the pursuit of non-ideal lung donors is warranted.

